# CONTEXTUALIZING THE RANGE OF MEDICATION-RELATED REGULATORY REQUIREMENTS IN ASSISTED LIVING

**DOI:** 10.1093/geroni/igad104.0212

**Published:** 2023-12-21

**Authors:** Sarah Dys, Lindsey Smith

**Affiliations:** Portland State University, Portland, Oregon, United States; Oregon Health & Science University, Portland, Oregon, United States

## Abstract

Prior research has investigated the severity and scope of medication-related deficiency citations; less is known about the specificity of regulatory requirements governing medication-related practices in assisted living (AL). We examine the variation of regulatory specificity for two common policies: medication record review and administration. We coded state requirements for 196 AL licenses linked to 33,246 ALs in the U.S for the presence of specific requirements, weighted for increasing precision (1-4), summed and divided by the maximum value to produce a score ranging from no specificity (0) to maximum specificity (1). Medication record review specificity includes: regularly scheduled review of resident medications generally (1), by AL staff (2), or by a licensed healthcare professional (3). Specificity of staff who administer oral medication includes: direct care staff permitted (1), certified nursing assistant or medication aide required (2), licensed practical/vocational nurse required (3), registered nurse required (4). Most facilities operate under licenses that only require general review of residents’ medications (62%) and 32% required medication review by a licensed healthcare provider. Most facilities’ licensure requirements permit direct care workers to administer medications (63%) and 16% require a licensed nurse to administer. One in six facilities have requirements that do not address who is permitted or required to administer oral medications. Overall, 36% of facilities operated under regulations that had an overall specificity score ( management and administration) of 0.5 or higher. While most ALs are licensed with low medication policy specificity, understanding regulatory expectations can contextualize and inform medication practices within these settings.

